# Development and validation of preconception care improvement scale (PCIS) in a resource-limited setting

**DOI:** 10.1186/s12884-021-04338-8

**Published:** 2022-01-11

**Authors:** Firanbon Teshome, Zewdie Birhanu, Yohannes Kebede

**Affiliations:** Department of Health, Behavior and Society, Faculty of Public Health, Jimma University, Jimma, Ethiopia

**Keywords:** PCIS, Validation, Pregnant women

## Abstract

**Background:**

Preconception care helps to close the gaps in a continuum of care. It is of paramount importance to reduce maternal and child adverse pregnancy outcomes, increase the utilization of services such as antenatal care, skilled delivery care, and post-natal care, and improve the lives of future generations. Therefore, a validated instrument is required. The purpose of this study was to develop and validate the preconception care improvement scale (PCIS) in a resource-limited setting.

**Methods:**

A mixed-method study was carried out from 02, March to 10, April 2019 in Manna district, Oromia region, Ethiopia to test the reliability and validity of the scale. Items were generated from literatures review, in-depth interviews with different individuals, and focused group discussions with women of reproductive age groups. A pretested structured questionnaire was used and a survey was conducted among 623 pregnant women in the district. The collected data were entered into EPI-data version 3.1 software and exported to SPSS version 23 software and data were analyzed for internal consistency and validity using reliability analysis and factor analysis.

**Results:**

The PCIS has 17 items loaded into six factors: Substance-related behaviors, screening for common non-communicable and infectious diseases, micronutrient supplementation and vaccination, seeking advice, decision and readiness for conception, and screening for sexually transmitted diseases. Factor analysis accounted for 67.51% of the observed variance. The internal consistency (Cronbach’s alpha) of the scale was 0.776. Diversified participants of the qualitative study and experts’ discussions assured the face and content validity of the scale. Factor loading indicated the convergent validity of the scale. Three of the PCIS subscale scores had a positive and significant association with the practice of preconception care and antenatal care visits, which confirmed the predictive validity of the scale.

**Conclusion:**

The PCIS exhibited good reliability, face validity, content validity, convergent validity, and predictive validity. Thus, the scale is valid and helps to improve preconception care, especially in resource-limited settings.

## Introduction

During the last four decades, efforts to improve pregnancy outcomes have mainly focused on antenatal care, skilled birth attendants and postnatal care [1]. However, the maternal and child mortality rates are still high. Evidence indicates that about 303,000 mothers died from pregnancy and childbirth-related causes and 99% of the deaths occurred in low and middle-income countries [2] This indicates that the world is far from achieving a sustainable development goal that aims to reduce the global maternal mortality ratio to less than 70 per 100,000 live births by the year 2030 [3]. The high prevalence of maternal and child mortality was mainly due to two main reasons: The first was due to the reason that women are too late to attend services like antenatal care and thus the initial critical period of the first 1000 days is frequently missed [4]. The second reason was due to a gap in the continuum of care (as the majority of women don’t receive care before conception). Evidence showed that globally less than 1/3rd of women of childbearing age visited health institutions and spoke with health care providers prior to pregnancy about their health status and its potential impact on pregnancy outcome [5].

Preconception care (PC) is a preventive, promotive or curative health care intervention provided to women of childbearing age before pregnancy and between subsequent pregnancies to improve pregnancy-related health outcomes for women, new-borns and children [6, 7]. It bridges the gap in the continuum of care and addresses pre-pregnancy health risks and health problems that could have negative maternal and fetal consequences [8, 9]. Several interventions can be provided during the preconception period. For instance, the findings of a systematic review showed that over 80 interventions have been recommended to be included in a PC package [1]. However, the package of PC interventions delivered in a particular setting depends on the local epidemiology, interventions already being delivered, and the resources in place to deliver additional interventions [5, 7]. Thus, prioritization based on needs and feasibility is important. In general, the recommended PC interventions are grouped into three main components: screening/ risk assessment, counseling/health promotion, and interventions/ management [1, 10].

The preconception period provides an opportunity to intervene earlier to optimize the health of potential mothers and to prevent risky behaviors. The majority of problems occurring during pregnancy can be addressed before women conceive [11]. Evidence has shown that PC interventions play a significant role in reducing maternal adverse pregnancy outcomes (abortion, anemia during pregnancy, maternal mortality) and neonatal and child adverse pregnancy outcomes (low birth weight, preterm birth, stillbirth, neonatal, and child mortality) through prevention of diseases, and timely identification and management of a number of modifiable factors that affect pregnancy outcomes [1, 7, 11]. In addition, PC also contributes to the social and economic development of families and communities [1, 7]. Indeed, it helps to increase the utilization of services such as antenatal care, skilled delivery care, and postnatal care [12] and improve the health of potential mothers, children, and future generations [11]. Thus, reliable and valid tools that enable the measurement of preconception health care are needed.

There are preconception health care tools from high-income countries, particularly from Canada [13–15]. However, to the best of our knowledge, no published work has validated the preconception care improvement scale from low-income countries. The components and mechanisms of delivery of preconception care need to be tailored to the realities of different countries depending on the local epidemiology, the interventions already being delivered, and the existing resources [5]. Therefore, this study aimed to develop and examine the reliability and validity of the PCIS in a resource-limited setting using a mixed-method approach. For many topics, review of the literatures yield dozens of published articles that can inform the process of developing the instrument. However, for some novel and rarely studied topics (such as improving preconception health care), the literature search may yield little relevance and hence qualitative approach is of paramount importance. In this study, a qualitative approach was used to generate items, explore how the target audiences comprehend, interpret the items, answer survey questions, identify potential problems that lead to response errors, comment on wording of the items and the overall format of the tool [16–19]. In addition, we used a quantitative approach to develop the scale, test the reliability and validity of the scale [19, 20].

## Methods

### Study Design and setting

A mixed-methods study was conducted from 02, March to 10, April 2019 in the Manna district, Oromia region, Ethiopia. The qualitative approach used in-depth interviews (IDIs) and focused group discussions (FGDs) with diverse individuals. A total of 623 pregnant women participated in the quantitative study.

### Phases and procedures

The scale development process involves complex and systematic procedures that require theoretical and methodological rigor [21, 22]. The PCIS was developed and validated by following a logical and structured approach that passed through three main phases: Item development, scale development and scale evaluation [19, 20, 23].

#### Phase 1: Item development

Item development is about coming up with the initial set of questions for an eventual scale. This phase involves identification of the domains and item generation, and consideration of face and content validity.

##### Identifying and defining Women’s PC improvement domains and items

Preconception care is a new concept in Ethiopia and there are no existing validated instruments that can adequately serve the same purpose. We focused on three iterative methods to identify the domains and pool of items that can measure each domain. First, we conducted review of relevant literatures. Accordingly, we identified three domains: behavioural, biomedical and social domains of preconception care. Additionally, the literatures review helped us to identify a pool of 23 potential items that can measure domains. Second, IDIs and FGDs were conducted. A total of 13 IDIs were conducted with women of different age groups, health extension workers, and health care providers of different professions. In addition to IDIs, 4 FGDs were held with women of reproductive age groups. Pregnant women, women who recently gave birth, and women planning to become pregnant participated in each focused group discussion. Semi-structured interview and FGD guides were developed to facilitate in-depth interviews and focused group discussions. The guides were co-designed with the target audience. It was prepared by elicitation with five women and two health care providers. In addition, a detailed discussion was made among the authors considering the research questions during the preparation of the guides. Details about the guides were explained in a separate published article [10]. Participants were invited to talk about their actual experiences regarding what women do for the sake of becoming pregnant.

A number of individuals participated in IDIs and FGDs, and ideas were gathered from different perspectives. However, the majority of the items elicited from IDIs and FGDs were similar to those obtained from the literatures review. Only 4 new items were obtained from the qualitative study (Table 1). Third, discussions with experts were conducted and the first version of an instrument on women’s PCIS which contained three domains and 27 items were constructed. All items were worded in statement form considering the local communities’ language and each item has three response options formatted as “Yes”, “No” and “Don’t know”. As much as possible, we tried to keep the items simple, straightforward, and followed the conventions of normal conversation (Table 1).

**Table 1:** Items generated from literatures review, in-depth interviews and focused group discussions

S/No	Items generated	Source
1	Q1: Screening for HIV/AIDS for the sake of becoming pregnant	Literatures, IDIs and FGDs
2	Q2: Screening for STI for the sake of becoming pregnant	Literatures, IDIs and FGDs
3	Q3: Screening for hypertension for the sake of becoming pregnant	Literatures, IDIs and FGDs
4	Q4: Screening for diabetes mellitus for the sake of becoming pregnant	Literatures, IDIs and FGDs
5	Q5: Screening for blood group for the sake of becoming pregnant	Literatures, IDIs
6	Q6: Screening for Hepatitis-b for the sake of becoming pregnant	Literatures, IDIs and FGDs
7	Q7: Screening for low blood for the sake of becoming pregnant	Literatures, IDIs and FGDs
8	Q8: Taking folic acid for the sake of becoming pregnant	Literatures
9	Q9: Taking iron or ferrous for the sake of becoming pregnant	Literatures
10	Q10: Taking Tetanus vaccine for the sake of becoming pregnant	Literatures and IDIs
11	Q11: Screening for obesity for the sake of becoming pregnant	Literatures, IDIs and FGDs
12	Q12: Consulting health workers for advice for the sake of becoming pregnant	Literatures, IDIs and FGDs
13	Q13: Having good nutrition and diet for the sake of becoming pregnant	Literatures, FGDs
14	Q14: Avoiding or cessation of drinking alcohol for the sake of becoming pregnant	Literatures, IDIs and FGDs
15	Q15: Avoiding or cessation of cigarette smoking for the sake of becoming pregnant	Literatures, IDIs and FGDs
16	Q16: Avoid or cessation of chewing khat for the sake of becoming pregnant	IDIs and FGDs
17	Q 17: Avoiding or cessation of drinking coffee for the sake of becoming pregnant	IDIs and FGDs
18	Q18: Avoiding or cessation of using cannabis or hashish for the sake of becoming pregnant	Literatures, IDIs and FGDs
19	Q19: Discussing with husband when to have a child for the sake of becoming pregnant	IDIs and FGDs
20	Q20: Stopping or removing family planning, ( if using) for the sake of becoming pregnant	IDIs and FGDs
21	Q21: Care before getting pregnant has benefit for baby	Literatures, IDIs and FGDs
22	Q22: Care before getting pregnant has a benefit for mothers	Literatures, IDIs and FGDs
23	Q23: Care before getting pregnant has a benefit for families	Literatures, IDIs and FGDs
24	Q24: Husband health condition matters for healthy conceptions	Literatures
25	Q25: Cares before pregnancy are obtained from home	Literatures and IDIs
26	Q26: Cares before pregnancy are obtained from communities	Literatures
27	Q27: Cares before pregnancy are obtained from health institutions	Literatures, IDIs and FGDs

##### ***Face and content validity***

Face validity is established when experts look at the items in the questionnaire and agree that the test is a valid measure of the concept which is being measured just at face value [24]. In the current study, to ensure face validity, the questionnaire was shown to three health promotion and health behaviour students. Indeed, during pre-testing of the draft instrument, 15 women were invited to comment on the clarity, simplicity, language, phrasing, and how well the questionnaire matched their experiences and invited for suggestions. Although all agreed on the questionnaire, some points were raised regarding local words and phrases, which were incorporated in the final version. Indeed, experts reviewed each item and modifications were made. Accordingly, to clearly identify preconception care from antenatal care and reduce confusion; experts decided to add the phrase “***for the sake of becoming pregnant***” to each item. Finally, the inputs obtained from different sources enabled researchers to confirm the face validity of the scale.

Content validity is the degree to which the sample of items, tasks, or questions on a test is representative of a defined universe or domain of content [24]. Content validity was assessed prior to the instrument being administered to the target population. To ensure that the content is valid, items were generated from many different sources (extensive literatures review, IDIs with different groups of individuals, and FGDs with diversified reproductive age group women). In addition, the adequacy, relevancy, and representativeness of the items generated to measure the developed domains were assessed by experts. After identifying a list of items to be validated, the authors consulted three experts on health communication and health behavior and two experts on reproductive health. The experts independently reviewed the items. Then, the authors and the multidisciplinary experts discussed each item, reached a consensus, and finally, the questionnaire and items were modified or accepted as it stands. This, allowed us to capture insights into the content validity. In this study, the content validity was not quantitatively assessed.

#### Phase 2: Scale development

This phase focuses on turning individual items into a harmonious and measuring construct. It involves the pretesting of questions, sampling and survey administration, item reduction, and extraction of factors.

##### ***Pre-testing Questions***

A pre-test was conducted in the Saka district among 15 pregnant women. Saka district is the neighbor of Manna district. Based on the findings obtained from the pre-test, some modifications were made to sequences, grammar, word choice, and how to conduct the interview with the target populations. During pre-testing, participants were invited for an opinion on whether they understand questions as developers intended and that respondents are able to answer in a manner that reflects their experience and assess the appropriateness of the questions to the target populations and the strength of the responses. Accordingly, all respondents suggested that the questions and aim of the instrument are clear. However, they suggested the need for rephrasing some items and word choice. Their feedbacks were incorporated into the final version of the tool.

##### Population, sampling, and survey administration

A total of 623 pregnant women (70 from urban and 553 from rural gandas) who lived in the Manna district at least six months prior to the study period participated in the main survey. The sample size was proportionally allocated to urban and rural gandas. The sampling frames of rural women were obtained from the family folder of the community health information system. For urban, since the family folder did not exist, a census was conducted to construct the sampling frame. The details were explained in the previously published articles, which were part of the current work [11, 25].

A total of 6 data collectors (4 clinical nurses and 2 BSc nurses) and 2 public health officers as supervisors were recruited for the main survey. The recruitment was based on their previous experience in data collection and fluency in the language of the community. Extensive training was provided for both data collectors and supervisors on the objectives of the study, data collection tools and collection processes. The details was explained in the previously published articles, which were part of the current work [11, 25].

#### Phase 3: Scale validation

##### ***Analysis***

Analyses were performed using the Statistical Package for the Social Sciences (SPSS) version 23. Principal component analysis (PCA) was performed for item reduction and extraction of factors. Prior to conducting the PCA, the assumptions were examined to determine whether it was appropriate to continue with the specific analysis. Accordingly, sampling adequacy was determined by Kaiser–Meyer–Olkin (KMO), which lies between 0 and 1 where a value of 1 indicates that each variable is perfectly predicted by the other variables. KMO > 0.5 is acceptable [26]. Bartlett’s test of sphericity helps to test for significant correlations among the variables for at least some of the variables. A P-value < 0.05 for Bartlett’s test of sphericity indicated a significant correlation among the variables and was acceptable. The acceptability of the two tests (KMO and Bartlett’s test of sphericity) indicated the suitability of the data for principal component analysis [26].

The extent to which scores on one item are related to scores on all other items on a scale, and the extent to which items on a scale assess the same content were assessed by inter-item correlation. Accordingly, items with very low correlations (< 0.30) were considered less desirable and deleted from the scale. In addition, items with a factor loading of < 0.4 were suppressed. Indeed, Items loaded on multiple factors with factor loading > 0.4 which were very close (< 0.1 difference) were removed from all the factors. However, items loaded on multiple factors with high factor loading showing significant differences across (≥ 0.1), retained on the factor with higher factor loading [27, 28].

We determined the number of components or factors based on: Kaiser Rule, the percentage of variation that is explained, the pattern of factor loadings, and the scree plot. Based on the Kaiser Rule [26], we retained the factors with an eigenvalue greater than 1.0. In addition, the variance explained by each sub-scale and scale was > 50%, which is acceptable [29]. Furthermore, researchers also observed the breaking point or elbow of Cattell’s scree plot, which helps to determine the number of factors [30].

## Psychometric Properties

**Reliability:** is the degree of consistency exhibited when a measurement is repeated under identical conditions. In this study, it was assessed using Cronbach’s alpha coefficients. The Cronbach’s alpha coefficient ranges from 0 to 1, and values between 0.7–0.9 were regarded as an acceptable threshold for reliability [31, 32].

**Validity:** is the extent to which instruments measure the latent dimension or construct it was developed to evaluate [33]. In this study, the researchers assessed face validity, content validity, convergent validity, and predictive validity. We explained *face and content validity* in phase1, item development. *Convergent validity* is the extent to which a construct measured in different ways yields similar results (e.g. self-report versus observation) [34]. It can be assessed through correlations between measures representing constructs that were hypothesized to be more strongly related according to prior expectations based on the theoretical relationships among constructs. In this study, convergent validity was confirmed based on the items score of factor loading and Bartlett’s test of sphericity. *Predictive validity* refers to the ability of the questionnaire to measure events, behavior, attitudes, or outcomes in the future [35]. In this study, logistic regression analysis was carried out and the predictive validity of the scale was estimated by examining the association between the sub-scale scores and behaviours (practice of preconception care and antenatal care visits). The practice of preconception care was dichotomized as “good or healthy PC practice” and “poor or unhealthy PC practice”. Antenatal care (ANC) visits for the current pregnancy were dichotomized into < 4 and ≥ 4 ANC visits. Details reported in separate published articles [8, 11].

### Ethical considerations

The ethical approval was obtained from the Institutional Review Board (IRB) ethics committee of Jimma University institute of health, approval code IHRPGD/356/19 on 01, March 2019. The permission was also sought from the Manna district health office and Ganda leaders. All the study participants were informed about the purpose of the study, their right to refuse at any time, and assured about the confidentiality of the information they provide. Written informed consent to participate in the study was obtained from all the study participants prior to the interview. For participants under 16 years or illiterate participants, written consent was obtained from their parents. All methods were performed in accordance with the relevant guidelines and regulations.

## Results

### Sociodemographic characteristics of the respondents

A total of 623 pregnant women participated, with a response rate of 98.0%. More than half, 352(56.5%) and 328(52.6%) of the respondents were in the age range of 25–34 years and had no formal education, respectively. A majority, 553(88.8%) of the respondents were living in rural areas. Nearly three-fourth, 462(74.2%) of the participants were housewives (Table 2).

**Table 2:** Socio-demographic characteristics of pregnant women in Manna district, Oromia region, Southwest Ethiopia, 2019 (*N* = 623)

Variable	Category	Frequency	Percent
Age of the respondents	15–24	196	31.5
	25–34	352	56.5
	35–49	75	12.0
Residence	Rural	553	88.8
	Urban	70	11.2
Religion	Muslim	583	93.6
	Orthodox	28	4.5
	Protestant	12	1.9
Ethnicity	Oromo	580	93.1
	Dawuro	21	3.4
	Amhara	14	2.2
	Other^a^	8	1.3
Educational level of the respondents	No formal education	328	52.6
	Primary education (1–8)	231	37.1
	Secondary education (9–12)	56	9.0
	Tertiary (college or university)	8	1.3
Main occupation of the respondents	Housewife	462	74.2
	Farmer	106	17.0
	Merchant	39	6.3
	Other^b^	16	2.6
Marital status	Married	618	99.2
	Other^c^	5	0.8

^a^Kaffa, Gurage and Silxe ^b^Student, Daily worker, Private employee, and Government employee ^c^Single and separated

### Principal Component Analysis: PCIS refinement

Correlation analysis and Explanatory factor analysis with subsequent three rounds of PCA validated a 17 items PCIS scale containing six components. First, a correlation analysis of 27 generated items was conducted. Of the 27 items, five were dropped due to a weak correlation. Before conducting the factor analysis, we checked for its assumptions and some parameters were fixed. Accordingly, the overall Kaiser–Meyer–Olkin was 0.76, which showed that the sample was adequate to carry out factor analysis. Bartlett’s test of sphericity was significant (*p* < 0.001), indicating that the correlation between the items was sufficiently large for factor analysis. The extraction was based on Eigenvalues, in which items with Eigenvalues > 1 were fixed. Varimax rotation was used to simplify the interoperability of the factor solution. Small variances with absolute value < 0.4 were suppressed and the remaining variances were sorted by size to display the coefficients from largest to smallest. Then, a series of three rounds of iterative exploratory factor analysis using PCA was carried out to identify a parsimonious list of factors that enable the improvement of preconception care. Each round involves deleting one or more items at a time and examining the remaining items (Summarized in Table 3).

**Table 3:** Summary of the three rounds of Principal component analysis

PCA rounds	Factors extracted	KMO	Bartlett’s test of sphericity	Items Retained	Total variance explained
Round 1	7	0.804	< 0.001	19 of 22 items	57.90%
Round 2	6	0.793	< 0.001	17of 19 items	64.77%
Round 3	6	0.760	< 0.001	17 items	67.51%

The first round of PCA of 22 items extracted seven factors with KMO = 0.804 and significant Bartlett’s test of sphericity (*p* < 0.001). During the first round of PCA, 3 items were removed due to low factor loading (< 0.4) and the total variance explained was 57.90%. Then, the second round PCA of 19 items extracted six factors with a total variance of 64.77%, with adequate sample size (KMO = 0.793), significant Bartlett’s test of sphericity (*p* < 0.001). During this round, 2 items were loaded on multiple factors with a factor loading difference of < 0.1 and thus removed from all the factors. Finally, a third-round PCA of 17 items extracted six factors with a total variance explained (VE = 67.51%), KMO = 0.76 and significant *P*-value (< 0.05) of Bartlett’s test of sphericity. During the third round of PCA, one item was loaded on multiple factors with factor loading > 0.4 and had significant differences (≥ 0.1). Thus, it was retained on the factor with a higher factor loading.

Correlation analysis and Varimax rotated three rounds of PCA produced a 17 items validated PCIS scale with six domains or factors. The extracted six factors were: Substance-related behaviours (4 items), screening for common non-communicable and infectious diseases (4 items), micro-nutrient supplementation and vaccination (3 items), seeking advice (2 items), decision and readiness for conception (2 items), and screening for sexually transmitted diseases (2 items) (Table 4).

**Table 4:** Varimax rotated scale components of preconception care improvement scale, Manna district, Oromia region, Ethiopia 2019 (*N* = 623)

Rotated Component/ factors loading score						
**Preconception care improvement scale**	**Component**					
	**1**	**2**	**3**	**4**	**5**	**6**
**Factor 1: Substance related behaviours**						
Q15: Avoiding or cessation of cigarette for the sake of becoming pregnant	0.870					
Q14: Avoiding or cessation of alcohol for the sake of becoming pregnant	0.858					
Q16: Avoid or cessation of chewing khat for the sake of becoming pregnant	0.707					
Q18: Avoiding or cessation of using cannabis for the sake of becoming pregnant	0.655					
**Factor 2: Screening for common non-communicable and infectious diseases**						
Q4: Screening for diabetes mellitus for the sake of becoming pregnant		0.803				
Q5: Screening for blood group for the sake of becoming pregnant		0.756				
Q3: Screening for hypertension for the sake of becoming pregnant		0.709				
Q6: Screening for Hepatitis b for the sake of becoming pregnant		0.645				
**Factor 3: Micro-nutrient supplementation and vaccination**						
Q9: Taking iron or ferrous for the sake of becoming pregnant			0.849			
Q8: Taking folic acid for the sake of becoming pregnant			0.833			
Q10: Taking Tetanus vaccine for the sake of becoming pregnant			0.598			
**Factor 4: Seeking advice**						
Q12: Consulting health workers for advice for the sake of becoming pregnant				0.802		
Q13: Having good nutrition and diet for the sake of becoming pregnant				0.800		
**Factor 5: Decision and readiness for conception**						
Q20: Stop or remove family planning, ( if user) for the sake of becoming pregnant					0.827	
Q19: Discussion with husband when to have a child					0.735	
**Factor 6:Screening for sexually transmitted diseases**						
Q1: Screening for HIV/AIDS for the sake of becoming pregnant						0.800
Q2: Screening for sexually transmitted disease for the sake of becoming pregnant						0.406

**Fig. 1:**
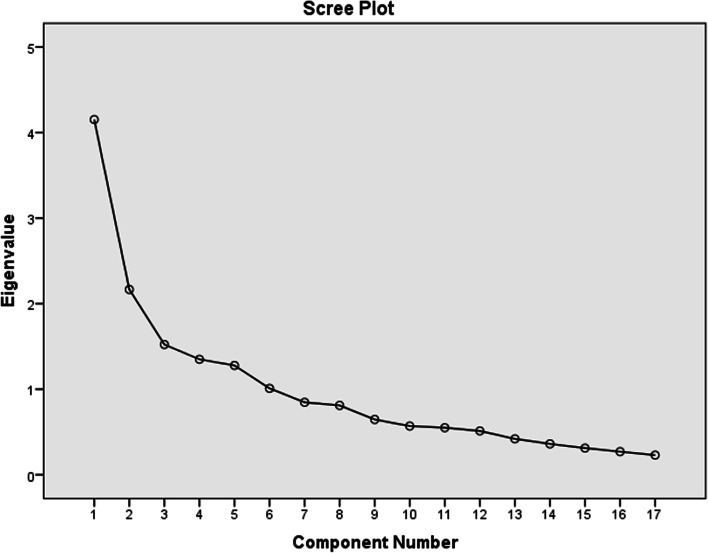
Scree plot of Eigenvalue and component number of 17-items PCIS, Manna district, Oromia region, Ethiopia 201

The scree plot of eigenvalues against the component number was used to determine the number of relevant components or factors retained in the PCA, by visualizing the magnitude of the variability associated with each component extracted by PCA (Fig. 1). Finally, the name was assigned for the factors considering the core idea explained by the predominant items in terms of factor loading in each sub-scale.

### Psychometric Properties

#### Reliability

Cronbach’s alpha for all items (PCIS) was 0.776. Cronbach’s alpha for sub-scales: Factor 1 (substance-related behaviors), factor 2(screening for common non-communicable and infectious diseases) and factor 3(micro-nutrient supplementation and vaccination) were 0.817, 0.780 and 0.761, respectively. All the calculated Cronbach’s alphas were in the acceptable range (0.7–0.9), indicating good reliability of the sub-scales and the scale. However, Cronbach’s alpha coefficient was not calculated for sub-scales with < 3 items (Table 5).

**Table 5:** Scale and subscales reliability analysis (*N* = 623)

Rotated scale components	Cronbach’s alpha
Substance-related behaviors	0.817
Screening for common non-communicable and infectious diseases	0.780
Micro-nutrient supplementation and vaccination	0.761
Seeking advice	a
Decision and readiness for conception	a
Screening for sexually transmitted diseases	a
PCIS	0.776

a = Cronbach’s alpha coefficient not calculated for sub-scales with fewer than three items

#### Validity

This study assessed the face validity, content validity, convergent validity, and predictive validity of the preconception care improvement scale. The face validity of the scale was assessed by experts and the target populations. Content validity was ensured by experts from different disciplines. In addition, a methodological rigor that included comprehensive literature reviews and involvement of diverse participants in IDIs and FGDs helped to give insight into the content validity of the scale (Details are described in Phase 1: Item development). In this study, the content validity was not quantitatively assessed. Convergent validity was examined by performing PCA with varimax rotation. The overall KMO was 0.76. Bartlett’s test for sphericity was significant (*p* < 0.001), indicating that the variables correlated with one another. Indeed, the factor loading of almost all items loaded on each factor was > 70%, which helps to assure the convergent validity of the scale (Table 3). Furthermore, as indicated in Table 6, Pearson correlation coefficients among the six extracted factors and the 17-item PCIS assessed the convergent validity. Accordingly, the convergence of the six subscales towards the PCIS ranged from *r* = 0.432 to 0.878, referring to a valid convergence score (> 0.4).

**Table 6:** Correlations of domains of preconception care improvement scale in Manna District, Oromia Region, Ethiopia, 2019 (*N* = 623)

Scale and sub-scales	SRB	SCNCD	MSV	SA	DRC	SSTD	PCIS
SRB	1						
SCNCD	0.512^a^	1					
MSV	0.479^a^	0.502^a^	1				
SA	0.382^a^	0.452^a^	0.443^a^	1			
DRC	0.414^a^	0.392^a^	0.436^a^	0.350^a^	1		
SSTD	0.455^a^	0.539^a^	0.383^a^	0.418^a^	0.454^a^	1	
PCIS	0.784^a^	0.878^a^	0.613^a^	0.568^a^	0.432^a^	0.548^a^	1

*SRB* Substance related behaviours, *SCCID* Screening for common non-communicable and infectious diseases, *MSV* Micro-nutrient supplementation and vaccination, *SA* Seeking advice, *DRC* Decision and readiness for conception, *SSTD* Screening for sexually transmitted diseases, ^a^Correlation is significant at the 0.01 level (2-tailed)

The predictive validity of the scale was examined by determining the association of subscales with practice of preconception care and antenatal care visits. Findings obtained from the regression analysis indicated that three of the PCIS subscales scores had a positive and significant association with the practice of preconception care and antenatal care visits, which assured the predictive validity of the scale (Table 7). Findings showed that the likelihood of understanding the need for screening for common non-communicable and infectious diseases during the preconception period was 1.7 times higher among women who had good practice of preconception care. Similarly, the odds of understanding the need to avoid substance-related behaviours during preconception care was 1.3 times higher among women who had a history of ≥ 4 ANC visits for the current pregnancy. Likewise, the likelihood of understanding the need for decision and readiness for conception during the preconception period was 2.3 folds among women with a history of ≥ 4 ANC visits for the current pregnancy (Table 7).

**Table 7:** Association between preconception improvement sub-scales and practice of preconception care and antenatal care visit (*N* = 623)

Sub-scales of PCIS	Practice of PC AOR (95% CI)	*P*—value	ANC visit AOR (95% CI)	*P*—value
Substance related behaviours	1.18 [0.92- 1.51]	0.185	1.26[1.08–1.48]	**0.004**
Screening for common non-communicable and infectious diseases	1.69 [1.26–2.27]	** < 0.001**	1.19[1.00–1.42]	0.055
Micro-nutrient supplementation and vaccination	1.07 [0.72–1.61]	0.731	1.05[0.76–1.44]	0.780
Seeking advice	1.18 [0.70–2.01]	0.536	1.15[0.85–1.55]	0.364
Decision and readiness and for conception	0.86 [0.47–1.56]	0.611	2.33[1.61–3.38]	** < 0.001**
Screening for sexually transmitted diseases	0.74 [0.39–1.39]	0.348	1.48[0.99–2.20]	0.053

## Discussion

In this study, we developed and validated a 17-items scale, named the preconception care improvement scale (PCIS). Six components of PCIS were extracted. Namely: substance-related behaviours; screening for common non-communicable and infectious diseases; micronutrient supplementation and vaccination; seeking advice; readiness and decision for conception, and screening for sexually transmitted diseases. The psychometric properties of the tool indicated good reliability, face validity, content validity, convergent validity, and predictive validity.

The process of development and validation of PCIS utilized standard procedures followed by most social, health, and behavioural scaling research or guides [19, 20, 34]. Accordingly, we started with item generation through extensive literatures reviews, IDIs, and FGDs with diverse individuals. The generated items were presented for experts’ panel discussion for evaluation. Then, the scale was developed through iterative processes by conducting a pre-test for drafted tool modifications, and the actual survey was conducted among the target population. Finally, the scale was examined for psychometric properties using reliability and factor analysis. During the PCA rounds, draft items with very weak correlation (*r* < 0.3), low factor loading (< 0.4), and double loadings at high factor loading but the insignificant difference (< 0.1) caused the removal of the items of the scales [36, 37]. A series of three rounds of PCA produced a 17 items PCIS with six factors which jointly accounted for 67.51% of the observed variance. Evidence showed that scales and sub-scales with a total variance explained > 50% were acceptable [29].

The first factor extracted was substance-related behaviors, indicating the need for advising women about avoiding or cessation of substance use before getting pregnant. This was similar to the preconception health behavior scale, hazardous substance factor [38]. The second factor produced was screening for common non-communicable and infectious diseases. This implies that women need to be checked for their wellness before becoming pregnant [25]. The third factor was micronutrient supplementation and vaccination. Supplementation of micronutrients such as folic acid is highly important. For instance, evidence showed that the prevalence of neural tube defects was decreased by 50–70% through supplementation of folic acid during the preconception period [39, 40]. The fourth extracted factor was seeking advice. This was similar to the preconception health behavior scale, which pointed out the acquisition of information [38]. This calls for the need to encourage women of childbearing age to visit health institutions and speak with health care providers prior to pregnancy about their health status and its potential impact on pregnancy outcomes. The fifth produced factor was decision and readiness for conception. This concept is very near to conception/pregnancy, which indicates the confirmation of their becoming pregnant. The final factor extracted was screening for sexually transmitted diseases, implying that women need to screen for locally prevalence sexually transmitted diseases including HIV/AIDS.

Finally, a 17 items PCIS with six factors was examined for reliability and validity. Reliability analysis showed that the internal consistency (Cronbach’s alpha) of the scale was 0.776 and in the acceptable range (0.7–0.9), indicating the scale was reliable [31]. Indeed, the PCIS was examined for different types of validity. The face validity and content validity of the scale were assured by conducting compressive literatures reviews, the involvement of diverse participants in IDIs and FGDs, and multidisciplinary experts’ panel discussion. This was supported by evidence obtained from different studies [19, 23, 41]. The PCIS also has predictive validity in that three sub-scales were significant predictors of the practice of preconception care and antenatal care visits. This finding was supported by studies from the Manna district, Ethiopia, which pointed out the association between common non-communicable diseases and antenatal care visits [11, 25]. This finding was also supported by a study from the USA [42]. This implies that increasing women’s awareness about the need of avoiding or cessations of substance use, screening for common communicable and infectious diseases, and their readiness for conception helps to increase their chance of getting subsequent services such as the practice of preconception care, antenatal care, skilled delivery and postnatal care [12]. Findings also showed that the construct validity of PCIS seems sufficiently secured. The evidence for the construct validity of the scale was obtained from factor analysis, which showed that the factor loading of almost all items loaded on each factor was > 70%. Studies pointed that factor loading scores of > 0.70 as having high convergent validity, which means that the items do not have information on factors other than the corresponding one [43]. Indeed, significant (*p* < 0.001) Bartlett’s test for sphericity indicated the variables correlated with one another, which was one evidence for convergent validity [44, 45]. The Pearson correlation coefficients among the six extracted factors and the 17-item PCIS showed the convergence of the six subscales towards the PCIS (*r* > 0.4) which indicated the convergent validity of the scale [46, 47]

This study has several strengths and limitations. A key strength of this study is that the PCIS has been validated in a large sample of participants. In addition, it was a community-based study that makes it a representation of the true population. Indeed, the comprehensive literatures review, the involvement of diverse participants in IDIs and FGDS, and the involvement of experts from different disciplines were the strengths of the scale. Furthermore, to the best of the authors’ knowledge, this was the first preconception improvement scale and it can pave the way for researchers to produce other tools. A study never ends without limitations. The potential limitation of this study was that content validity was not quantitatively assessed. The unavailability of PCIS evidence overtime was another critical limitation of this study. Indeed, this study was conducted among pregnant women and thus there was a chance of recall bias. In addition, interviewer bias may also have occurred. Hence, this study shall be tested again broadly in a diverse population.

## Conclusion

A 17 items PCIS developed with six components (substance-related behaviors, screening for common non-communicable and infectious diseases, micronutrient supplementation and vaccination, seeking advice, decision and readiness for conception, and screening for sexually transmitted diseases) was found to be a valid and reliable scale. Having a validated tool appropriate for the population is really important to bring the desired behavior change. Therefore, PCIS helps to improve preconception care, especially in resource limed settings. The authors recommend researchers to test the scale again among different women of reproductive age groups such as women planning to become pregnant and currently married women.

## Data Availability

The data of the study are available from the corresponding author upon reasonable request.

## Abbreviations

ANC: Antenatal care
FGD: Focused group discussion
IDI: In-depth interview
KMO: Kaiser–Meyer–Olkin
PC: Preconception care
PCA: Principal component analysis
PCIS: Preconception care improvement scale
SPSS: Statistical package of social science
